# Structural and functional responses of plant communities to climate change‐mediated alterations in the hydrology of riparian areas in temperate Europe

**DOI:** 10.1002/ece3.3973

**Published:** 2018-03-26

**Authors:** Annette Baattrup‐Pedersen, Annemarie Garssen, Emma Göthe, Carl Christian Hoffmann, Andrea Oddershede, Tenna Riis, Peter M. van Bodegom, Søren E. Larsen, Merel Soons

**Affiliations:** ^1^ Department of Bioscience Aarhus University Silkeborg Denmark; ^2^ Department of Biology Utrecht University Utrecht The Netherlands; ^3^ Section for Ecology and Biodiversity Swedish University of Agricultural Sciences Uppsala Sweden; ^4^ Department of Bioscience Aarhus University Aarhus Denmark; ^5^ Institute of Environmental Sciences Leiden University Leiden The Netherlands

**Keywords:** climate change, drought, flooding, lowland, plant, trait, vegetation

## Abstract

The hydrology of riparian areas changes rapidly these years because of climate change‐mediated alterations in precipitation patterns. In this study, we used a large‐scale in situ experimental approach to explore effects of drought and flooding on plant taxonomic diversity and functional trait composition in riparian areas in temperate Europe. We found significant effects of flooding and drought in all study areas, the effects being most pronounced under flooded conditions. In near‐stream areas, taxonomic diversity initially declined in response to both drought and flooding (although not significantly so in all years) and remained stable under drought conditions, whereas the decline continued under flooded conditions. For most traits, we found clear indications that the functional diversity also declined under flooded conditions, particularly in near‐stream areas, indicating that fewer strategies succeeded under flooded conditions. Consistent changes in community mean trait values were also identified, but fewer than expected. This can have several, not mutually exclusive, explanations. First, different adaptive strategies may coexist in a community. Second, intraspecific variability was not considered for any of the traits. For example, many species can elongate shoots and petioles that enable them to survive shallow, prolonged flooding but such abilities will not be captured when applying mean trait values. Third, we only followed the communities for 3 years. Flooding excludes species intolerant of the altered hydrology, whereas the establishment of new species relies on time‐dependent processes, for instance the dispersal and establishment of species within the areas. We expect that altered precipitation patterns will have profound consequences for riparian vegetation in temperate Europe. Riparian areas will experience loss of taxonomic and functional diversity and, over time, possibly also alterations in community trait responses that may have cascading effects on ecosystem functioning.

## 1 INTRODUCTION

In temperate regions, such as Northern and Central Europe, climate change‐associated alterations in precipitation patterns, with higher than average precipitation and less snow accumulation during winter and lower than average precipitation during summer, likely mediate significant alterations in the hydrological characteristics of lowland streams. In winter and early spring, an increase in the frequency, magnitude, and duration of flow events will occur (Karlsson, Sonnenborg, Seaby, Jensen, & Refsgaard, 2015; van Roosmalen, Sonnenborg, & Jensen, 2009; Thodsen et al., 2014), whereas the frequency and duration of drought periods are expected to increase during summer (Andersen et al., 2006; Christensen & Christensen, 2007). Higher temperatures will likely intensify deficits in water budgets during summer through enhanced evaporation and evapotranspiration, both of which will intensify water stress (Douville et al., 2002). Furthermore, higher temperatures may extend the active growth period of plants as growth may start earlier in spring and continue for a longer time, thereby possibly exacerbating the effects of flooding and droughts on natural ecosystems (Zwicke et al., 2013).

Climate change effects on the structural and functional properties of riparian ecosystems remain to be more fully elucidated. Increasing awareness of the importance of wetlands for a number of ecosystem services such as flood protection, water purification, water availability via groundwater recharge, and biodiversity has spurred new studies into the functioning of wetlands in a changing climate (see Catford et al., 2013; Kominoski et al., 2013; Garssen, Verhoeven, & Soons, 2014; Garssen, Baattrup‐Pedersen, Voesenek, Verhoeven, & Soons, 2015 for an overview). Most of the studies conducted so far investigate the effects of climate changes on riparian community composition with focus on the response of a single species or restricted species assemblages (Catford et al., 2013; Garssen et al., 2014, 2015). A recent extensive review of plant community responses showed that prolonged flooding and increased inundation depth of riparian areas trigger significant shifts in species composition that may lead to either increased or decreased riparian species richness, depending on the environmental characteristics of the areas (Garssen et al., 2015).In Garssen et al. (2015), species richness was observed to generally decline at flooded sites in nutrient‐rich catchments and at sites previously exhibiting relatively stable hydrographs (for instance rain‐fed lowland streams; see e.g., Beltman, Willems, & Güsewell, 2007; Baattrup‐Pedersen, Jensen, et al., 2013), whereas an increase in species richness was detected at flooded sites in dry areas (e.g., in deserts and semi‐arid climate regions where many streams are intermittent; see e.g., Stromberg, Hazelton, & White, 2009; Horner, Cunningham, Thomson, Baker, & Mac Nally, 2012). In contrast, almost all studies of the effects of increased drought episodes on riparian plant community responses have shown a decline in species richness, particularly for herbaceous species (e.g., Stromberg, Bagstad, Leenhouts, Lite, & Makings, 2005; Westwood, Teeuw, Wade, Holmes, & Guyard, 2006; reviewed in Garssen et al., 2014). A > 30‐day drought period threatens the survival of many species and usually entails a strong reduction in riparian plant biomass, and a high drought intensity (i.e., a 3–4 cm water table decline per day) may impair riparian seedling survival, thereby producing relatively rapid changes in riparian species composition (Garssen et al., 2014).

The functional trait characteristics of plant species will likely determine whether the species are able to survive under changed environmental conditions (Cornwell & Ackerly, 2009; Jung et al., 2014). Hence, trait‐based predictions of the response of riparian communities to climate change are valuable. In contrast to taxonomic approaches, trait‐based methods enable generalizations (i.e., identification of common responses) to be made across regions (Catford et al., 2013; Diaz et al., 2004). A wide range of traits can be used to describe the responses of species to their environment, and different traits may capture different aspects of resource use, habitat requirements, and stress responses (e.g., Suding et al., 2006; Thuiller, Albert, Dubuis, Randin, & Guisan, 2010). Traits related to life form characteristics, growth forms, growth rates, photosynthetic pathways, leaf morphology, and chemistry have all been used to identify plant responses to environmental conditions as they affect species growth, survival, and reproductive output (de Bello & Mudrak, 2013; Violle et al., 2007; Westoby & Wright, 2006).

In this study, we explored the effects of an experimentally altered hydrology on the taxonomic and functional trait characteristics of the vegetation and deposited seeds in riparian areas. To increase the predictive potential, we used a large‐scale experimental approach in which we manipulated water levels to disentangle the effects of specific environmental changes from co‐occurring environmental characteristics that may otherwise blur the responses (see Ackerly, 2004; Douma, Bardin, Bartholomeus, & Bodegom, 2012; Wright, Reich, & Westoby, 2003). An additional strength of this approach was that the direct large‐scale water level manipulations applied permits creation of groundwater–surface water interactions resembling those likely to occur in riparian areas under current and expected rates of climatic change. To identify cross‐regional consistent patterns responses in the vegetation, the experimental sites were located in Denmark, Germany, and the Netherlands. In some parts of the sites, we experimentally increased flooding in the winter/spring and in other parts of the sites we increased droughts in summer.

**Figure 1:**
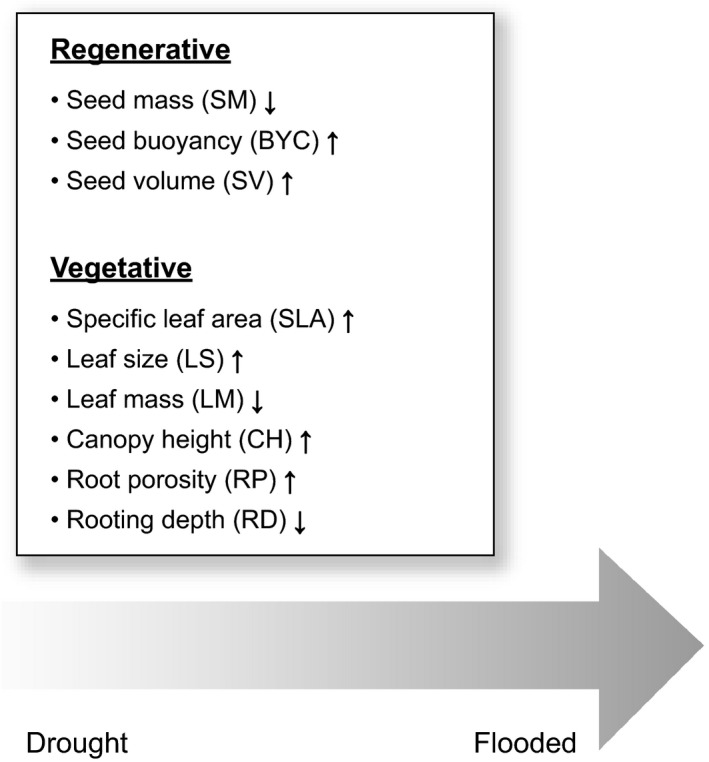
Hypothesized changes in community trait composition moving from drought to flooded conditions. Arrows indicate whether a trait is expected to increase or decrease with increased flooding, with an expectation of the opposite response to drought

We analyzed regenerative traits and vegetative traits that we expected would change under altered hydrological conditions (Figure 1). The selection of traits was based on theoretical considerations: Hydrological alterations are likely to affect traits associated with the ability to increase the water uptake and/or conserve water as well as traits associated with the ability to survive conditions with water surplus (Douma et al., 2012; Hough‐Snee et al., 2015). The vegetative traits included leaf traits (specific leaf area, size, and mass), root traits (rooting depth and porosity), and canopy (maximum height) that may show an adaptive response to cope with an altered hydrology. Under drought conditions, we expected that the abundance of species with extensive rooting depths and species with dense stems, small and thick leaves, and low specific leaf areas would increase in abundance. These traits can serve to maximize water uptake and at the same time reduce water loss as the rate of transpiration generally decreases with declining specific leaf area and leaf mass (Wright et al., 2005; Swenson & Enquist, 2007; Poorter & Markesteijn, 2008; Douma et al., 2012; Figure 1). Under flooded conditions, we expected that the abundance of species with traits associated with the ability to lower the metabolic activity (the “quiescence strategy”) or avoid unfavorable conditions (the “escape strategy”; Bailey‐Serres & Voesenek, 2008) would increase. Therefore, we anticipated that the abundance of tall species would increase as these have more easy access to atmospheric oxygen than short species. Additionally, we expected that species able to form porous roots or aerenchyma in adventitious roots to facilitate oxygen transport to the apical root zone (Armstrong, Brandle, & Jackson, 1994) would increase in abundance, as these traits can be critically important to maintain the exchange of gas under flooded conditions (Bailey‐Serres & Voesenek, 2008; Garssen et al., 2015). We also considered regenerative traits associated with the ability to disperse under drought and flooded conditions, respectively, including seed mass, volume, and buoyancy. Specifically, we expected that species with a high seed mass would decline in abundance with enhanced flooding concomitantly with an increase in species with a high seed buoyancy and volume, reflecting the adaptive value of producing low mass but high volume buoyant seeds that can disperse efficiently by water (Douma et al., 2012).

The specific hypotheses tested were that flooding and drought mediate the following: (1) a decline in the taxonomic and functional diversity of traits and (2) a shift in the mean functional trait values as depicted in Figure 1. These responses will expectedly be strongest in near‐stream areas where the hydrological alterations are most pronounced and will intensify over time. Additionally, it was tested if (3) the taxonomic diversity and functional diversity of the seed pool were higher in flooded areas than in drought areas as the regional species pool may contribute to diversity through species dispersal by water (i.e., hydrochory; Nilsson, Brown, Jansson, & Merritt, 2010).

## 2 MATERIALS AND METHODS

### 2.1 Experimental setup

**Figure 2:**
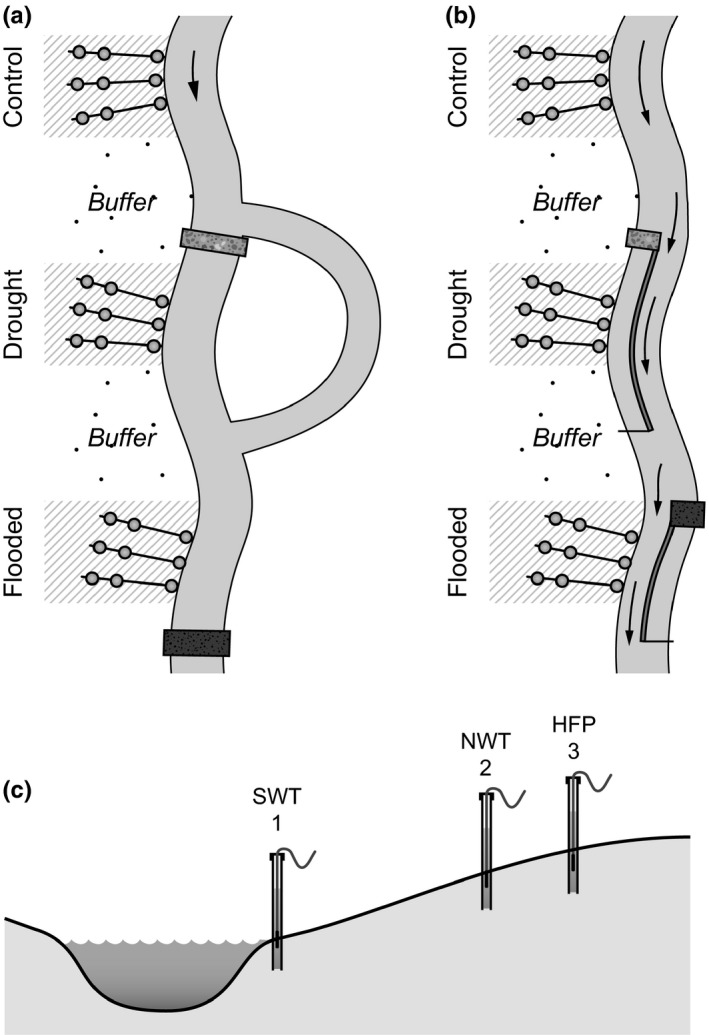
A schematic presentation of the experimental setup applied in our study. The control section is situated upstream of the drought and flooded sections with buffers in‐between. Flooding was created by constructing dams (marked as bars on the figure) to obstruct the water flow in the main channels. (a) In Denmark, a lateral dam of sandbags was constructed across the stream channel. (b) In Germany and the Netherlands, longitudinal dams were built within the channel, which together with a lateral dam across the channel obstructed the water flow in the main channel. (c) The position of the sample transects within the experimental sections. The first piezometer was placed just above the summer water table (position 1), the second piezometer just above the normal winter water table (position 2) and the third at the high end of the floodplain (position 3). The circles indicate the position of the piezometers along each transect

Four riparian areas situated along streams in Denmark (Sandemandsbækken 56.158507 N, 9.496120 E; Voel Bæk 56.195846 N, 9.703932 E), Germany (Boye 51°58′61.1″N, 6°91′10.01″E), and the Netherlands (Groote Molenbeek 51°39′17.32″N, 6°03′59.47″E) were selected for the experiment (Table 1). The four streams varied in mean discharge from 0.03 to 1.73 m^3^/s. This was, however, not considered problematic as our sampling effort was focused on covering the natural features of the stream‐riparian gradient at the study sites irrespective of size. That is, the sampling covered a gradient from the water table of the stream under summer base flow conditions to the high end of the floodplain where only extreme events lead to flooding (Figure 2).

**Table 1:** Study site characteristics

Site	Sandemandsbæk	Boye	Voel Bæk	Groote Molenbeek
Catchment area (km^2^)	0.07	3.40	7.57	183.56
Grassland (%)	0.16	0.31	0.02	0.43
Forest (%)	0.43	0.11	0.03	0.00
Urban (%)	0.05	0.15	0.04	0.07
Agriculture (%)	0.25	0.42	0.90	0.45
Wetlands (%)	0.11	0.00	0.00	0.00
Water (%)	0.00	0.01	0.00	0.00
Mean discharge (m^3^/s)	0.03	0.08	0.06	1.73

The length of the experimental areas was 150 m, whereas width varied depending on the extent of the stream‐riparian gradient. The areas were divided into three sections: a control section, a winter/spring flooded section, and a summer drought section. These hydrological treatments were selected to mimic hydrological changes in Europe as predicted by IPCC (2007). The riparian areas had not been exposed to floodings prior to the experiment and comprised seminatural grassland communities with only herbaceous species.

The control sections were situated upstream of the manipulated sections with buffer areas in‐between (Figure 2). Flooding was created by constructing dams in the streams to obstruct the water flow in the main channels. In Denmark, a lateral dam made of sandbags was established across the stream channel (Figure 2a), while in Germany and the Netherlands, longitudinal dams were built within the channel, which together with a lateral dam across the channel obstructed the water flow in the main channel (Figure 2b). The constructed dams were used to create a 6‐week flooding of the adjacent riparian areas (from March to mid‐April) in 2011, 2012, and 2013, where the strongest responses were expected to occur in the final year of sampling given that the areas have been subject to manipulation for several years. However, in 2013, flooding was delayed in Denmark due to ice cover and lasted from the end of April to mid‐June. In Denmark, summer droughts were created by digging a ditch, which together with a lateral dam in the main channel diverted part of the water flow from the main channel, resulting in a lowered water table within the experimental areas (Figure 2a). In Germany and the Netherlands, a longitudinal dam was constructed across the stream channel, which together with a lateral dam across the channel obstructed the water flow adjacent to the experimental area, thereby lowering the water table (Figure 2b). The drought experiment was conducted in 2011, 2012, and 2013 from the end of June to September (approximately 10 weeks) at all sites except Boye where strong groundwater seepage prevented reduction in the water table in the drought section.

Within each section, three sample transects were established perpendicular to the stream from the channel and upwards in the riparian areas (Figure 2). The length of the sample transects varied among the study sites in order to represent a gradient from the lowest water table of the stream under summer base flow conditions to the highest point of the stream valley potentially flooded by surface water during extreme winter floods (Figure 2c). To determine the hydrology of the control, drought, and flooded sections, a total of nine piezometers were installed within each section (three along each sample transect). The first piezometer was placed close to the stream, just above the normal summer water table in the stream, that is normally not flooded during summer but occasionally during winter floods (position 1; Figure 2c). The second piezometer was placed just above the normal winter water table that is normally not flooded in either summer or winter (position 2; Figure 2c). The third piezometer was placed at the highest point of the floodplain that was rarely flooded and, if so, only during extreme winter flooding events (once every 100 years; position 3; Figure 2c).

### 2.2 Characterization of hydrology and vegetation

The water table depths were measured at least four times during the experimental periods in each experimental year (at the start of the experiment, after 2 weeks, after 4 weeks, and at the end of the experiment). Mean values of water table depths are given in Table 2. Positive values indicate that flooding occurred; the more positive the values, the higher the flooding depths. Similarly, negative values indicate that the water table is situated below the surface, and the more negative the values, the deeper the water table. Close to the streams (position 1), the flooding treatment prolonged the duration of winter flooding and increased the depth of flooding, whereas the drought treatment generally lowered the groundwater table during the treatment period (Table 2). Further away from the stream at position 2, the flooding treatment resulted in occasional winter floodings during the treatment period, whereas the drought treatment lowered the groundwater table (Table 2). Farthest away from the stream (position 3), the flooding treatment resulted in overall higher groundwater tables during the treatment period, whereas the drought treatment lowered the groundwater table (Table 2).

**Table 2:** Means and *SE* of groundwater table depths measured in piezometers at least four times during each experimental run (at the start of the experiment, after 2 weeks, after 4 weeks, and at the end of the experiment). Positive values indicate that the water table was situated above the ground surface, and negative values indicate that the water table was situated below the ground surface. The piezometers were placed along a hydrological gradient. The first sampling point was at the lowest water table of the stream during summer base flow conditions (SWT). The second sampling point was just above the normal winter water table that is normally not flooded in either summer or winter (position 2). The third sampling point was at the highest point up the stream valley that could be flooded by surface water during extreme winter floods (position 3)

Site	Treatment	Position	Groundwater, mean (cm)	Groundwater, *SE*
Sandemandsbækken	Control	1	−10.35	1.68
		2	−22.35	1.73
		3	−16.59	1.32
	Drought	1	−18.86	1.44
		2	−26.75	1.89
		3	−20.93	2.45
	Flooded	1	1.34	1.90
		2	−0.77	2.27
		3	−26.43	1.07
Voel	Control	1	−10.07	0.94
		2	−16.13	1.01
		3	−29.36	1.56
	Drought	1	−35.23	1.73
		2	−49.35	2.20
		3	−56.10	2.39
	Flooded	1	1.10	1.79
		2	−0.50	1.77
		3	−24.28	1.63
Boye	Control	1	−8.79	1.81
		2	−9.96	2.13
		3	−22.74	3.36
	Flooded	1	13.70	2.10
		2	−0.18	3.68
		3	−30.12	2.12
Groote Molenbeek	Control	1	−5.27	3.09
		2	−15.87	2.53
		3	−21.52	3.48
	Drought	1	−8.72	1.78
		2	−33.00	2.14
		3	−37.75	3.14
	Flooded	1	13.55	4.62
		2	1.29	2.89
		3	−4.50	1.39

Vegetation surveys were conducted during the growing season (June–September). Percentage coverage was estimated for all vascular species in a total of 27 plots (50 × 50 cm^2^) per site for each treatment. These were positioned with three plots next to each of the three piezometers in each of the three transects. Species composition was recorded according to the Braun‐Blanquet method (1928), adjusted by Barkman, Doing, and Segal (1964). In the two Danish sites, an additional 27 bare plots were established with three plots next to each of the three piezometers in each of the three transects in order to follow the establishment of the vegetation under the new hydrological settings during the experimental period. These were created by removing the existing vegetation and the topsoil followed by deposition of 15 cm mixed sand and peat. To avoid ingrowth of nearby plants, the plots were delineated using 15‐cm‐wide plastic bands that were vertically inserted into the soil.

Vegetation data were converted to Ord% scale (coverage ranges from 0.5 to 140) according to Van der Maarel (2007) for a cover‐based interpretation of the Braun‐Blanquet scale (Braun‐Blanquet, 1928). Seed traps consisting of 25 × 22.5 cm artificial mats with plastic bristles (Astroturf^®^) were placed and secured near the square plots used for vegetation surveys. Seeds were collected in 2011 in both control, flooded, and drought areas during the 6 weeks of experimental flooding and 10 weeks of experimental drought. The mats were removed from the field immediately after the experimental period and taken to the laboratory where they were stored in plastic bags in the dark at 4°C before processing. The processing involved extraction of deposited material by flushing the seed traps with water, followed by wet sieving the deposits to remove fine silt and clay. The material was then dried at 70°C for 48 hr after which intact seeds were visually identified from the dried material, manually removed, and determined to species level with the use of the “Digital seed atlas of the Netherlands” (Cappers, Bekker, & Jans, 2006).

### 2.3 Diversity indices and community‐weighted means of plant traits

All diversity and trait indices were calculated for each vegetation type based on Ord% values (van der Maarel 2007). We calculated taxon richness and Shannon diversity as indices of taxonomic diversity. Traits were allocated to the encountered species based on information available in the LEDA database (Kleyer & Bekker, 2008) and literature cited in Douma et al. (2012). We selected traits describing both seed (SM, BYC, SV; Table 3) and adult (SLA, LS, LM, CH, RP, RD; Table 3) plant characteristics expected to respond to an altered hydrological regime as described in the introduction (Figure 1). The number of species with trait information and the total abundances of these species are given in Table 3. We calculated functional divergence (FDvar) and community‐weighted means (CWMs) when the abundance of species with trait information was above 65%, thereby precluding specific leaf area, root porosity, and rooting depth (Table 3). The abundance limit represented a balance between on the one hand to have as many traits as possible integrated in the analyses to obtain insight into the functional response of the plant community to climate change‐related alterations in the hydrology of the areas, and on the other hand to keep the estimation bias low (Borgy et al., 2017). FDvar and CWMs were calculated for each trait according to Lavorel et al. (2007).

**Table 3:** Explanations of the traits used to characterize the riparian plant communities. Traits were derived from the LEDA database (Kleyer & Bekker, 2008) and from literature cited in Douma et al. (2012). The percentage of species with trait information was calculated as the number of species with trait information and as the abundance of species with trait information (in brackets). Three traits were excluded from the analyses (SLA, RD, RP) as the abundance of species with trait information was below 65%

Trait name	Unit	Category	% species with trait information
Seed buoyancy (BYC)	%	Seed	64 (65)
Seed mass (SM)	Mg	Seed	75 (78)
Seed volume (SV)	mm^3^	Seed	68 (73)
Specific leaf area (SLA)	mm^2^/mg	Adult	52 (55)
Leaf size (LS)	mm^2^	Adult	64 (70)
Leaf mass (LM)	Mg	Adult	62 (68)
Canopy height (CH)	M	Adult	74 (77)
Root porosity (RP)	%	Adult	31 (53)
Rooting depth (RD)	M	Adult	37 (65)

A response ratio (Δ*r*) (Osenberg, Sarnelle, & Cooper, 1997) for each diversity and trait metric was also calculated using mean values of three sample plots for each of the three sampling transects for each position as:

$$\Delta r=\ln\left(\frac{\mathrm{Nt}}{\mathrm{Nc}}\right)$$

where *Nc* is the mean metric value at the control site and *Nt* is the metric value for the treatment (flooded or drought). Response ratios allowed us to assess the general effects of the two treatments on riparian plant diversity and trait composition across the four streams.

### 2.4 Data analyses

All analyses described in this paragraph were conducted using the statistical software R (R Core Team 2014), package vegan (Oksanen et al., 2014). Canonical correspondence analysis (CCA) (function *cca*) followed by permutational ANOVAs (function *anova.cca* with maximum permutations set to 9999) was performed to assess differences in plant community composition between treatments (control, drought, flooding), type of vegetation (seed, existing vegetation, bareplot), and year (2011, 2012, 2013). To estimate the unique effect of a single predictor (i.e., treatment, type of vegetation, and year), the variation in plant community composition explained by the other predictors was always partialled out (i.e., included as covariables) in the ANOVAs. We also assessed which traits were significantly associated with differences in plant community composition between treatments by fitting trait vectors (describing the relative abundance of traits in each plot; i.e., CWMs) onto the CCA ordination using the function *envfit*. The *envfit* function finds the direction in the ordination space toward which each trait vector changes most rapidly and to which it is maximally correlated with the ordination configuration. The significance of the trait vectors was determined by a permutation test (*n* = 999).

To assess the general effects of the treatments across the study streams, we combined the yearly estimates into a single effect size measurement and tested whether the response ratios (Δr) of taxonomic diversity, trait diversity, and CWMs differed significantly from zero (i.e., higher or lower than zero) using two‐sided *t* tests. The yearly response ratio estimates were combined by a weighted average using the variance for year as the weight. *T* tests were performed separately for each vegetation type (seed, existing vegetation, bareplot). A significant result was interpreted as a consistent and detectable change in the metric value in the control site versus the treated (flooded or dry) site across the investigated streams.

## 3 RESULTS

**Figure 3:**
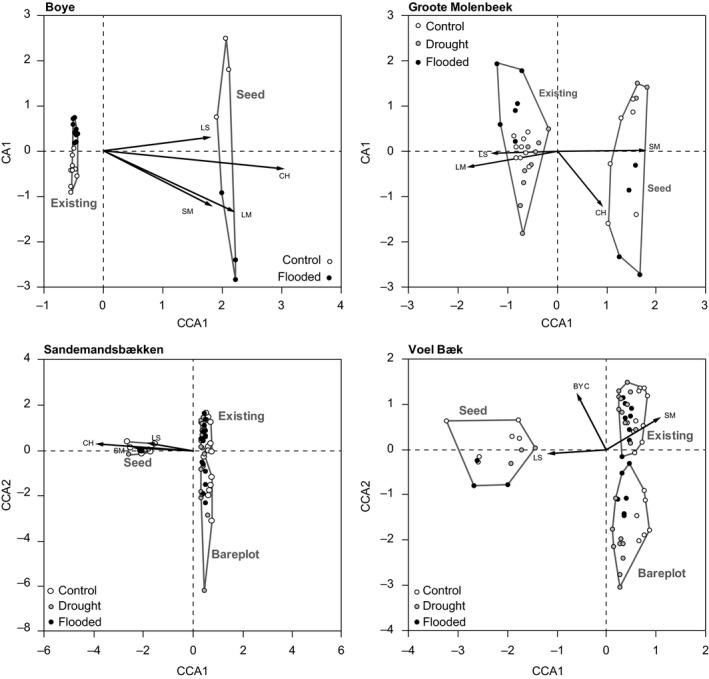
Ordination plots of the canonical correspondence analyses (CCAs) of plant species composition within each riparian area (Boye, Groote Molenbeek, Sandemandsbækken, and Voel Bæk). In the CCAs, species composition was constrained by the type of vegetation (seed, existing, and bareplot), whereas the variation in species composition explained by treatment (flood, drought, control) and year (2011, 2012, 2013) was partialled out. Traits significantly associated with the CCA axes (*p* < .05) are plotted onto the ordination

**Figure 4:**
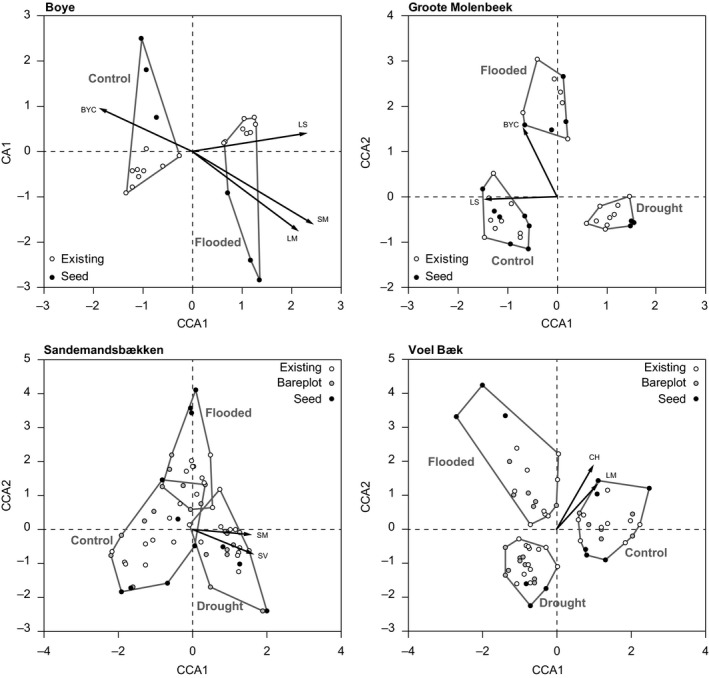
Ordination plots of the canonical correspondence analyses (CCAs) of plant species composition within each stream (Boye, Groote Molenbeek, Sandemandsbækken, and Voel Bæk). In the CCAs, species composition was constrained by treatment (flood, drought, control), whereas the variation in species composition explained by type of vegetation (seed, existing, and bareplot) and year (2011, 2012, 2013) was partialled out. Trait vectors significantly associated with the CCA axes (*p* < .05) are plotted onto the ordination

There were large variations in species composition among the four study sites regarding both type considered (i.e., seed pool, bare plot, and existing vegetation), treatment applied (i.e., control, drought, and flooding), and time of sampling (i.e., 2011, 2012 and 2013; Figures 3 and 4; Table 4). The effects of the applied treatment on the compositional patterns in the experimental areas were significant for both the seed pool and the existing vegetation (Figures 3 and 4; Table 4). Several of the traits used to describe the functional characteristics of the vegetation were associated with the main gradients in taxonomic composition (Tables 5 and 6), suggesting that they captured important underlying mechanisms responsible for the observed compositional changes.

**Table 4:** Summary statistics of the ANOVAs of the canonical correspondence analyses where species composition was constrained by treatment, type, or year. The variation of the other parameters was always partialled out (i.e., included as covariables) in the ANOVAs to enable estimation of the unique effect of a single parameter

Constraint	Covariables	Study site	*X* ^2^	*F* (*df*)	*Pr* (>*F*)
Treatment	Type; Year	Boye	0.352	2.571 (1.21)	0.005
		Groote Molenbeek	0.537	2.847 (2.34)	0.005
		Voel	0.377	2.976 (2.58)	0.005
		Sandemand	0.432	2.682 (2.58)	0.005
Type	Treatment; Year	Boye	0.909	6.647 (1.21)	0.005
		Groote Molenbeek	0.642	6.798 (1.34)	0.005
		Voel	0.680	5.409 (2.58)	0.005
		Sandemand	0.842	5.221 (2.58)	0.005
Year	Treatment; Type	Boye	0.352	1.224 (2.20)	0.079
		Groote Molenbeek	0.409	2.084 (2.34)	0.005
		Voel	0.224	1.696 (2.58)	0.005
		Sandemand	0.269	1.614 (2.58)	0.005

**Table 5:** Summary statistics of the envfit analyses where trait vectors (CWMs) were fitted to the ordination axes of the canonical correspondence analyses (CCAs). Summary statistics of the correlation between trait vectors and the first two ordination axes are shown. In the CCAs, plant species composition was constrained by the type of vegetation, while treatment and year were included as covariables (i.e., the variation in plant composition explained by treatment and year was partialled out)

Trait	Boye			Groote Molenbeek			Sandemandsbæk			Voel Bæk		
	CCA1	CA1	*r* ^2^	CCA1	CA1	*r* ^2^	CCA1	CCA2	*r* ^2^	CCA1	CCA2	*r* ^2^
BYC	−0.07	1.00	.05	0.80	0.60	.06	−0.53	0.85	.07	−0.44	0.90	.21**
SM	0.78	0.63	.30****	−0.52	−0.85	.08	0.95	0.33	.00	0.84	0.54	.18**
SV	0.42	0.91	.10	−0.62	−0.79	.02	0.50	−0.87	.02	0.54	0.84	.06
LS	0.99	0.17	.32**	−1.00	−0.03	.21*	−0.99	0.12	.12*	−1.00	−0.07	.15*
LM	0.85	−0.52	.63**	−0.98	−0.19	.40***	0.58	0.81	.01	−0.89	−0.46	.07
CH	0.99	−0.13	.88***	0.60	−0.80	.26**	−1.00	0.08	.45***	−0.68	0.73	.08****

***p < .001, **p < .01, *p < .05.

**Table 6:** Summary statistics of the envfit analyses where trait vectors (CWMs) were fitted to the ordination axes of the canonical correspondence analyses (CCAs). Summary statistics of the correlation between trait vectors and the first two ordination axes are shown. In the CCAs, plant species composition was constrained by treatment, while the type of vegetation and year were included as covariables (i.e., the variation in plant composition explained by treatment and year was partialled out)

Trait	Boye			Groote Molenbeek			Sandemandbæk			Voel Bæk		
	CCA1	CA1	*r* ^2^	CCA1	CA1	*r* ^2^	CCA1	CCA2	*r* ^2^	CCA1	CCA2	*r* ^2^
BYC	−0.89	0.46	.25	−0.41	0.91	.32	0.66	0.75	.07	−0.02	1.00	.07
SM	−0.36	0.93	.14	0.67	−0.75	.02	1.00	−0.09	.14*	0.94	−0.35	.01
SV	0.53	0.85	.11	−0.23	−0.97	.02	0.91	−0.40	.17**	−0.70	−0.72	.07
LS	0.98	0.18	.31*	−1.00	−0.04	.24*	0.99	−0.11	.03	−0.15	0.99	.05
LM	0.77	−0.63	.43**	−0.78	−0.63	.15****	−0.81	−0.59	.04	0.64	0.77	.11*
CH	0.54	−0.84	.02	−0.83	0.55	.13****	0.83	0.55	.05	0.47	0.88	.16**

***p < .001, **p < .01, *p < .05.

### 3.1 Existing vegetation

**Figure 5:**
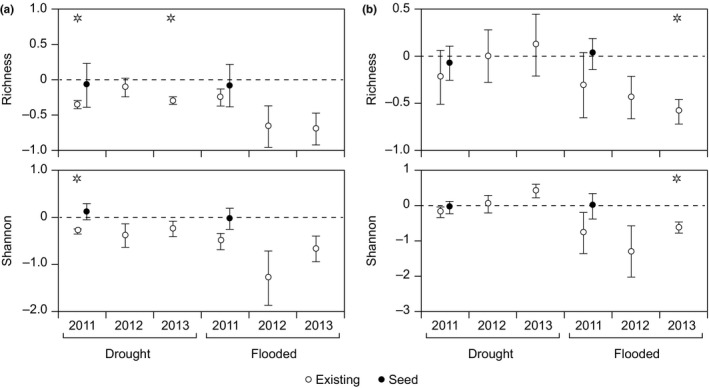
Average response ratios (±1 *SE*) of taxonomic diversity (richness and Shannon diversity) in plots positioned close to the stream channel just above the normal summer water table (position 1; a) and in plots situated just above the normal winter water table (position 2; b). No significant changes in richness or diversity occurred further up the floodplain, position 3, following the applied drought and flooding treatment. Open symbols (existing) comprise data for the vegetation surveys, whereas closed symbols (seed) comprise data for the seed trap surveys. The color of the asterisk indicates the type of vegetation differing significantly from zero (i.e., black asterisk = seed, white asterisk = existing)

Applying response ratios, we detected consistent changes among study sites for both the taxonomic and functional composition of the plant communities. In accordance with the first hypothesis, we observed that both species richness and Shannon diversity were negatively affected by drought and flooding and that the response varied with distance from the streams (Figure 5). At position 1, the richness and diversity of the existing vegetation declined in response to drought the first year after initiating the treatment (i.e., the response ratio was significantly lower than zero), and richness was still lower after 3 years of treatment (Figure 5). Further away from the streams at position 2, we observed a decline in species richness and diversity, but the response was only significant after 3 years of flooding (Figure 5).

**Figure 6:**
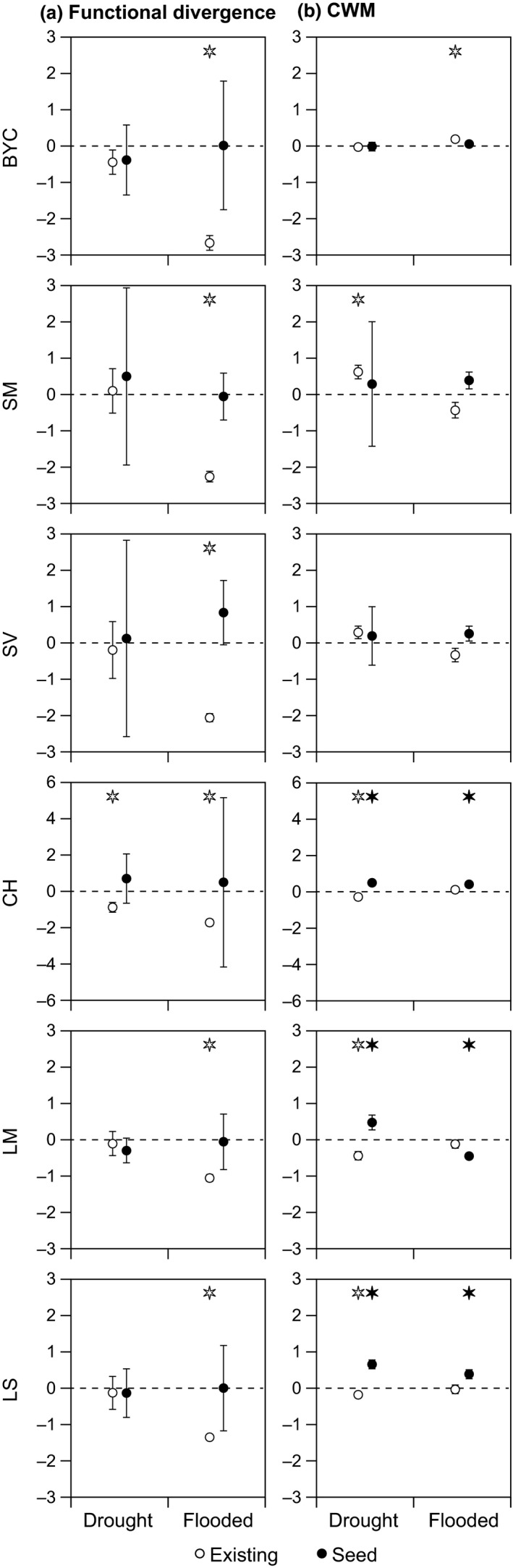
Average response ratios (±1 *SE*) of functional trait diversity (FDis) (a) and trait composition (CWMs) (b) in plots positioned close to the stream channel just above the normal summer water table (position 1). When a response ratio is significantly different from zero, this is indicated with an asterisk above the error bar (*p* < .05). Open symbols (existing) comprise data for the vegetation surveys, whereas closed symbols (seed) comprise data for the seed trap surveys. The color of the asterisk indicates the type of vegetation differing significantly from zero (i.e., black asterisk = seed, white asterisk = existing). Note that the scale for FDis for CH is different in comparison with the other traits

**Figure 7:**
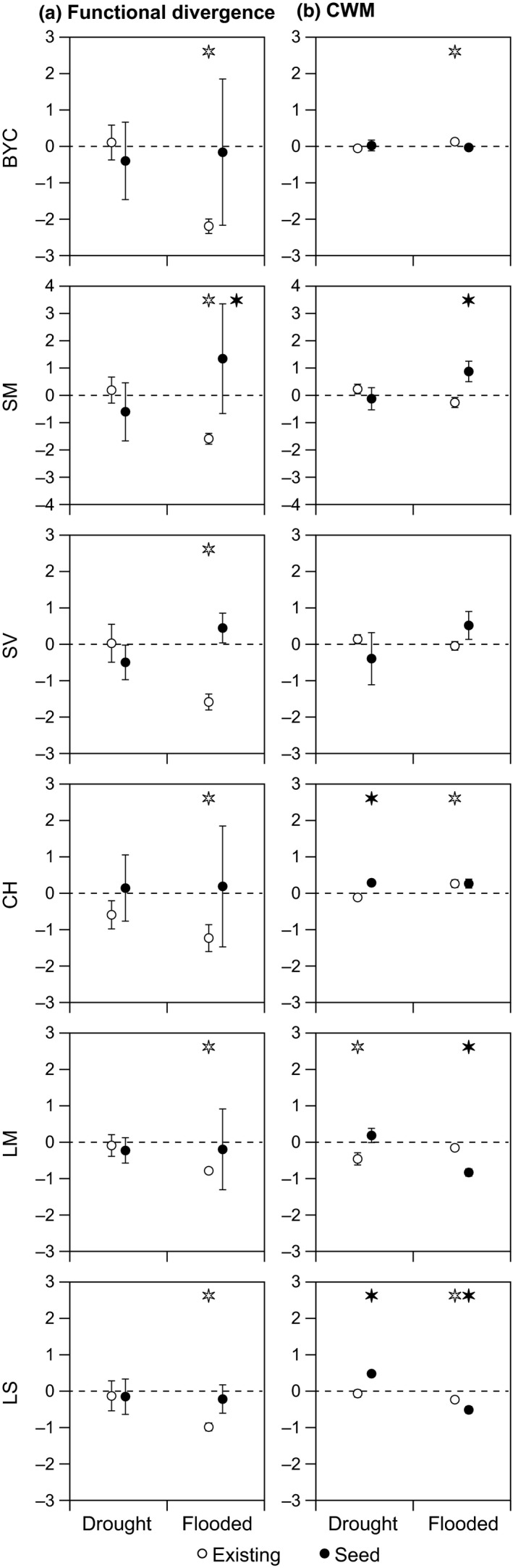
Average response ratios (±1 *SE*) of functional trait diversity (FDis) (a) and trait composition (CWMs) (b) in plots positioned just above the normal winter water table (position 2). When a response ratio is significantly different from zero, this is indicated with an asterisk above the error bar (*p* < .05). Open symbols (existing) comprise data for the vegetation surveys, whereas closed symbols (seed) comprise data for the seed trap surveys. The color of the asterisk indicates the type of vegetation differing significantly from zero (i.e., black asterisk = seed, white asterisk = existing. Note that the scale for FDis for SM is different in comparison with the other traits

**Figure 8:**
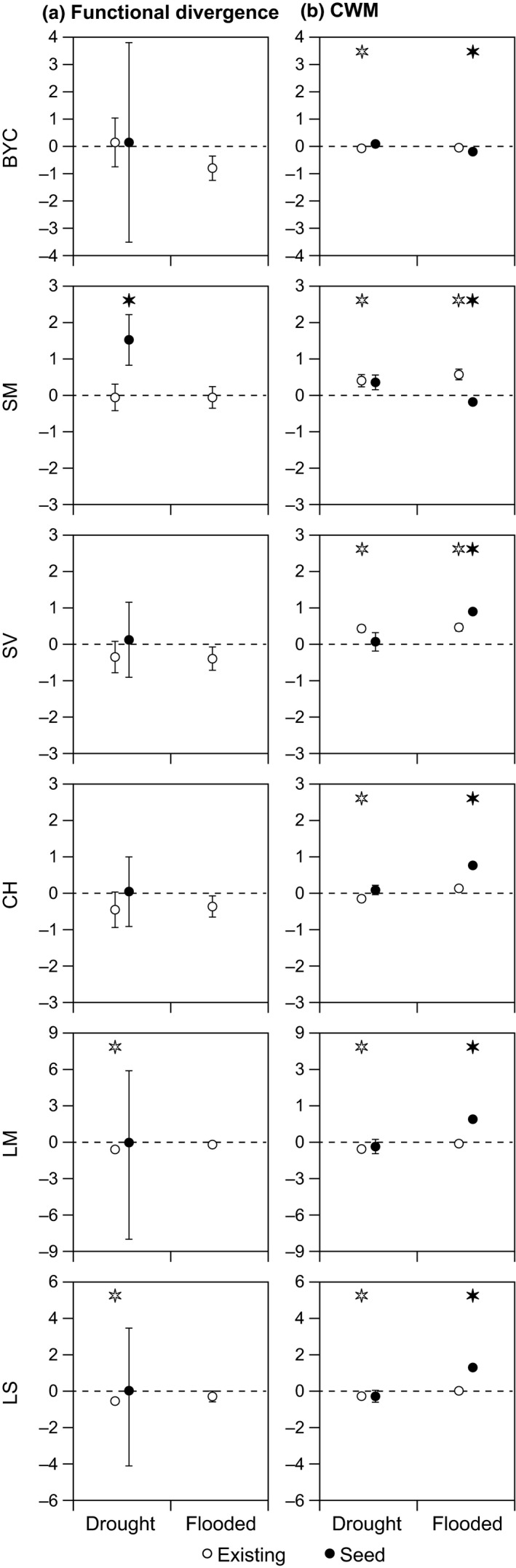
Average response ratios (±1 *SE*) of functional trait diversity (FDis) (a) and trait composition (CWMs) (b) in plots positioned at the high end of the floodplain (position 3). When a response ratio is significantly different from zero, this is indicated with an asterisk above the error bar (*p* < .05). Open symbols (existing) comprise data for the vegetation surveys, whereas closed symbols (seed) comprise data for the seed trap surveys. The color of the asterisk indicates the type of vegetation differing significantly from zero (i.e., black asterisk = seed, white asterisk = existing). Note that the scale for FDis for BYC, LM, and LS is different in comparison with the other traits

In accordance with the second hypothesis, we also identified consistent changes in the functional diversity of the existing vegetation in particular in response to flooding (Figures 6a, 7a, and 8a). Close to the streams, at positions 1 and 2, we observed that the functional diversity of all traits declined in response to 3 years of flooding (BYC SM, SV, CH, LM, and LS; Figures 6a and 7a), whereas the functional diversity of CH declined in response to 3 years to drought but only at position 1 (closest to the stream). Farthest away from the streams at position 3, we observed a decline in the functional diversity of two traits (LM and LS) in response to drought (Figure 8a).

In accordance with the second hypothesis, we also observed consistent changes in the mean functional trait (CWM) values of the existing vegetation in response to the applied treatments and, as demonstrated by the diversity patterns, the response varied with distance from the streams (Figures 6b, 7b, and 8b) and generally followed the predicted patterns (see Figure 1). Close to the streams, at position 1, BYC‐CWM increased in response to flooding and SM‐CWM increased in response to drought (Figure 6b) but, in contrast to our expectations, SV‐CWM declined in response to flooding. Further away from the stream, at position 2, BYC‐CWM and CH‐CWM increased in response to flooding and LS‐CWM declined (Figure 7b), but in contrast to our expectations, LM‐CMW declined in response to drought (Figure 7b). Farthest away from the stream at position 3, we observed an increase in SV‐CWM in response to flooding, also confirming our expectations (Figure 8b), but SM‐CWM increased which was in contrast to our expectations (Figure 8b). We also observed several significant changes in the trait composition of the community in response to drought at position 3 (BYC, SM, CH, LS, SV, LM) and for the majority of the traits, these changes were as predicted (BYC, SM, CH, LS; Figure 8b).

### 3.2 Seed pool

As opposed to our third hypothesis, we did not find a significant increase in the taxonomic richness or diversity of the seed pool in response to flooding (Figure 5; ANOVA; *p* > .05), but we observed an increase in functional diversity but only for SM at position 2 (Figure 7a). Instead, we observed several changes in the trait value of the seed pool in response to flooding (CH, LM, LS at position 1; SM, LM, LS at position 2; BYC, SM, SV, CH, LM, LS at position 3) and drought (CH, LM, LS at position 1; CH, LS at position 2) and most of these changes followed the predicted pattern (Figure 1) particularly close to the stream.

## 4 DISCUSSION

### 4.1 Taxonomic and functional diversity response

We found significant effects of flooding and drought on the species composition of both the vegetation and the seed pool in all study areas. Between‐study site variability was also prominent, and this is likely due to local differences in soil characteristics and/or hydrological conditions among the study sites that influence the effects of hydrological alterations on the riparian vegetation (Garssen et al., 2015). Despite the observed between‐study site variability, consistent patterns were also detected in response to hydrological changes. In particular, we observed a decline in both the taxonomic and functional diversity of the plant communities. The decline in taxonomic diversity in response to drought was only evident near the streams, probably reflecting that the experimental areas were already well drained and consequently less affected by the experiment (Table 2), whereas the negative impacts of flooding on species diversity were more pronounced (although only significant after 3 years of flooding). This finding may indicate that fewer species were able to tolerate flooding within the area compared with the number of species able to tolerate (relatively mild) drought and/or that dispersal constraints were higher for species adapted to flooded conditions. Our findings are in line with those of Ström, Jansson, Nilsson, Johansson, and Xiong (2011) where soil monoliths were transplanted to areas subjected to different flooding intensities within the riparian zone of a boreal river. Species diversity increased rapidly in monoliths transplanted to higher elevations (i.e., less flooding) over the course of the 6‐year field study, while species diversity in monoliths transplanted to lower elevations (i.e., more flooding) declined rapidly (Ström et al., 2011).

Functional diversity also responded to the altered hydrological settings, in particular in proximity to the streams. We observed a significant decline in the functional diversity of all traits, indicating that the range of successful strategies displayed under the new hydrological settings was restricted. Our finding lends support to previous studies suggesting that strong abiotic filters constrain the range of species mean trait values that can exist within the community, leading to a convergent trait distribution (Bernard‐Verdier et al., 2012; Jung, Violle, Mondy, Hoffmann, & Muller, 2010; Weiher, Clarke, & Keddy, 1998). In line with our observations for taxonomic diversity, also functional diversity responded more strongly to flooding than drought, indicating that flooding poses a more severe stress on the riparian community in temperate regions (Fraaije, Braak, Verduyn, Verhoeven, & Soons, 2015; Fraaije, Braak, Verduyn, Breeman, et al., 2015). The loss of functional diversity (1–2 years) may influence resource use efficiency within the systems, with cascading effects on ecosystem functioning (Díaz & Cabido, 2001). Further studies are, however, needed to explore this topic, with special emphasis on how climate change‐mediated alterations in hydrological extremes in combination with a higher degree of unpredictability in the occurrence of these affect ecosystem functioning.

### 4.2 Community functional trait response

The loss of functional diversity was also reflected in the mean trait response of the riparian plant community. We observed a consistent increase in the mean trait value of seed buoyancy in response to flooding, indicating that the fraction of species adapted to flooded conditions increased in the area. This finding is in accordance with Ozinga, Bekker, Schaminee, and Van Groenendael (2004) who, based on a classification of dispersal traits of ca. 900 species from different types of communities, found a highly significant correlation between the position of species along a wetness gradient and the frequency of morphological adaptations to hydrochory. This pattern has later been confirmed also for riparian and aquatic plant communities (van den Broek, van Diggelen, & Bobbink, 2005). As opposed to the findings of Douma et al. (2012), however, we did not observe a declining seed mass with enhanced buoyancy and seed density therefore seems to be a relatively poor predictor of seed buoyancy.

For the vegetative CWMs, we observed fewer consistent changes in comparison with those previously reported to respond to an altered hydrology (Bernard‐Verdier et al., 2012; Jung et al., 2010; Mommer, De Kroon, Pierik, Bögemann, & Visser, 2005; Violle et al., 2011; Voesenek, Colmer, Pierik, Millenaar, & Peeters, 2006). There may be several, nonmutually exclusive, explanations to the less consistent response of trait CWMs to the contrasting hydrological settings in our study. First, different adaptive strategies for different species may co‐occur in a community, which may partly explain the relatively weak response observed when comparing the mean trait value of single traits (Bernard‐Verdier et al., 2012; Douma et al., 2012). For example, some species may have small and thin leaves that facilitate oxygen uptake during submergence (Banach et al., 2009; Nielsen & Sand‐Jensen, 1989), enabling them to survive under flooded conditions, whereas other species may avoid flooded conditions by elongating their shoots, thereby accessing atmospheric oxygen (Voesenek, Rijnders, Peeters, Van de Steeg, & De Kroon, 2004) as also observed in our study. Second, intraspecific variability was not considered for any of the traits in this study, which may have weakened community responses (Albert, Grassein, Schurr, Vieilledent, & Violle, 2011; Jung et al., 2010). For example, many species can elongate shoots and petioles that enable them to survive shallow, prolonged flooding (e.g., Chen et al., 2009), but such abilities will not be captured when applying mean trait values. Third, we only followed the communities for 3 years after the change in hydrological settings. Altered hydrological conditions will likely mediate fast exclusion of species intolerant of these changes, whereas the establishment of new species relies on their dispersal and establishment within the areas. Therefore, a delay in the response of mean trait values of the community to changed habitat conditions may occur (Oddershede, Svenning, & Damgaard, 2015; Sandel et al., 2010), reflecting progressive filling of available niches within the community, eventually leading to stronger trait convergence (Helsen, Hermy, & Honnay, 2012; Roscher, Schumacher, Gerighausen, & Schmid, 2014). This delay may be stronger in existing vegetation than in bare plots where colonization and environmental filtering may occur rapidly (Fraaije, Braak, Verduyn, Verhoeven, et al., 2015; Fraaije, Braak, Verduyn, Breeman, et al., 2015) as also seen in the bare plots in our study, which differed significantly in species composition from the existing vegetation. Finally, we did not have traits for all species found in the areas, and the results regarding the response of community‐weighted trait means should therefore be treated with caution.

### 4.3 Seeds

We expected to find functionally more diverse seed pools in the flooded areas than in the drought areas, reflecting that hydrochory can introduce seeds from an upstream species pool in addition to seeds that may enter from the local species pool by wind and/or animal dispersal. Furthermore, earlier investigations have shown that seed deposition in flooded areas is highly dependent on flow patterns and microtopography within the areas and that the amount of seeds deposited coincides with the drift line in flooded areas (Nilsson & Grelsson, 1990; Riis, Baattrup‐Pedersen, Poulsen, & Kronvang, 2014). We therefore expected to find the highest diversity at intermediate distance from the streams. However, our study did not confirm this expectation as the functional diversity was unaffected by flooding. This finding indicates that species arriving by water may not be more functionally diverse than those arriving by other means of dispersal. This interpretation is supported by previous studies reporting that species dispersed by hydrochory are often those already locally abundant (Brederveld, Jähnig, Lorenz, Brunzel, & Soons, 2011; Soomers et al., 2011) and that flooding in itself may not be sufficient to increase species richness in grassland vegetation upon restoration of more natural flooding conditions (Baattrup‐Pedersen, Riis, & Larsen, 2013; Baattrup‐Pedersen, Dalkvist, et al., 2013; Bissels, Holzel, Donath, & Otte, 2004).

## 5 CONCLUSIONS

We observed large study site variability in plant community responses to the hydrological conditions of our experiment, regarding both drought and flooding. We did, however, identify consistent patterns in the taxonomic and functional responses of plant communities to the altered hydrological settings. Both taxonomic diversity and functional diversity were generally negatively affected by flooding and to some extent also by drought. These findings indicate that the range of successful strategies declined due to the altered hydrological settings. The loss in functional diversity was also reflected in the mean trait response of the riparian community but fewer significant and consistent changes appeared in response to the altered hydrological conditions. This might reflect a combination of the existence of several strategies within the vegetation to cope with the altered hydrological settings and a delay in the mean trait response due to a slow and progressive filling of available niches. Taken together, our results demonstrate that even though it is difficult within a 3‐year time frame to predict general effects of extreme hydrological conditions on riparian vegetation characteristics across large regions, the observed losses in diversity likely affect ecosystem functioning by reducing niche complementarity with possible cascading effects on resource use efficiency.

## CONFLICT OF INTEREST

None declared.

## AUTHORS CONTRIBUTIONS

ABP, AG, CCH, and MS designed the study, EG and SEL conducted the statistical analyses, AO and TR assisted in the field campaigns, and PMvD provided a number of traits for the species. ABP wrote the manuscript and all authors contributed to its finalization.
